# Extensive Thrombosis in a Severe COVID-19 Patient Despite Prophylactic Low Molecular Weight Heparin

**DOI:** 10.7759/cureus.11152

**Published:** 2020-10-25

**Authors:** Sherif Elkattawy, Sahitya Posimreddy, Nirmal Guragai, Mirette Habib, Fayez Shamoon

**Affiliations:** 1 Internal Medicine, Rutgers-New Jersey Medical School/Trinitas Regional Medical Center, Elizabeth, USA; 2 Cardiology, Saint Joseph's University Medical Center, Paterson, USA; 3 Cardiology, Trinitas Regional Medical Center, Elizabeth, USA

**Keywords:** covid, rv thrombus, anticoagulation

## Abstract

D-dimer >1 mcg/L has been shown to be an independent predictor of mortality, and experts from China have recommended starting prophylactic doses of anticoagulation in severe coronavirus disease 2019 (COVID-19) unless contraindicated. We present a case of extensive intravascular thrombosis in an otherwise healthy patient with severe COVID-19 disease despite prophylactic anticoagulation.

## Introduction

Severe acute respiratory syndrome coronavirus 2 (SARS-CoV-2) has inflicted over 35 million lives worldwide with the death toll surpassing 1 million lives as per the World Health Organization (WHO), 7.4 million of which are US civilians with a death toll over 200,000. The clinical manifestations of the virus are diverse and most patients seem to have a good prognosis, however those with underlying comorbidities tend to have coagulation dysfunctions in addition to typical symptoms (cough, fever, shortness of breath). About 50% of patients with COVID-19 are accompanied by elevated D-dimer levels during disease progression, and this proportion is as high as 100% in death cases [1]. The level of D-dimer in critically ill patients is significantly higher than that in mild patients, and some patients suddenly deteriorate during treatment, or even sudden death. This suggests that COVID-19 patients, especially those with severe symptoms, have a higher risk of thrombosis [2].

This case report discusses a middle-aged Hispanic female with COVID-19 who was found to have bilateral venous duplex with right ventricular thrombus.

## Case presentation

History of presentation 

A healthy 42-year-old Hispanic female with no significant past medical history presented to the emergency department for worsening shortness of breath and dry cough preceded by a four-day history of subjective fever associated with body aches and headache. Patient took Tylenol with minimal relief. Review of systems was negative for abdominal pain, diarrhea, chest pain, orthopnea and dizziness. Her initial vitals on admission were temperature 101 F, blood pressure 138/84, heart rate 104, respiratory rate of 24 with saturation 91% on room air and 95% on 2L nasal cannula. Physical examination was pertinent for diffuse rhonchi. Electrocardiogram was unremarkable.

Differential diagnosis

Influenza, community-acquired Pneumonia and COVID-19 were considered.

Investigations

**Figure 1:**
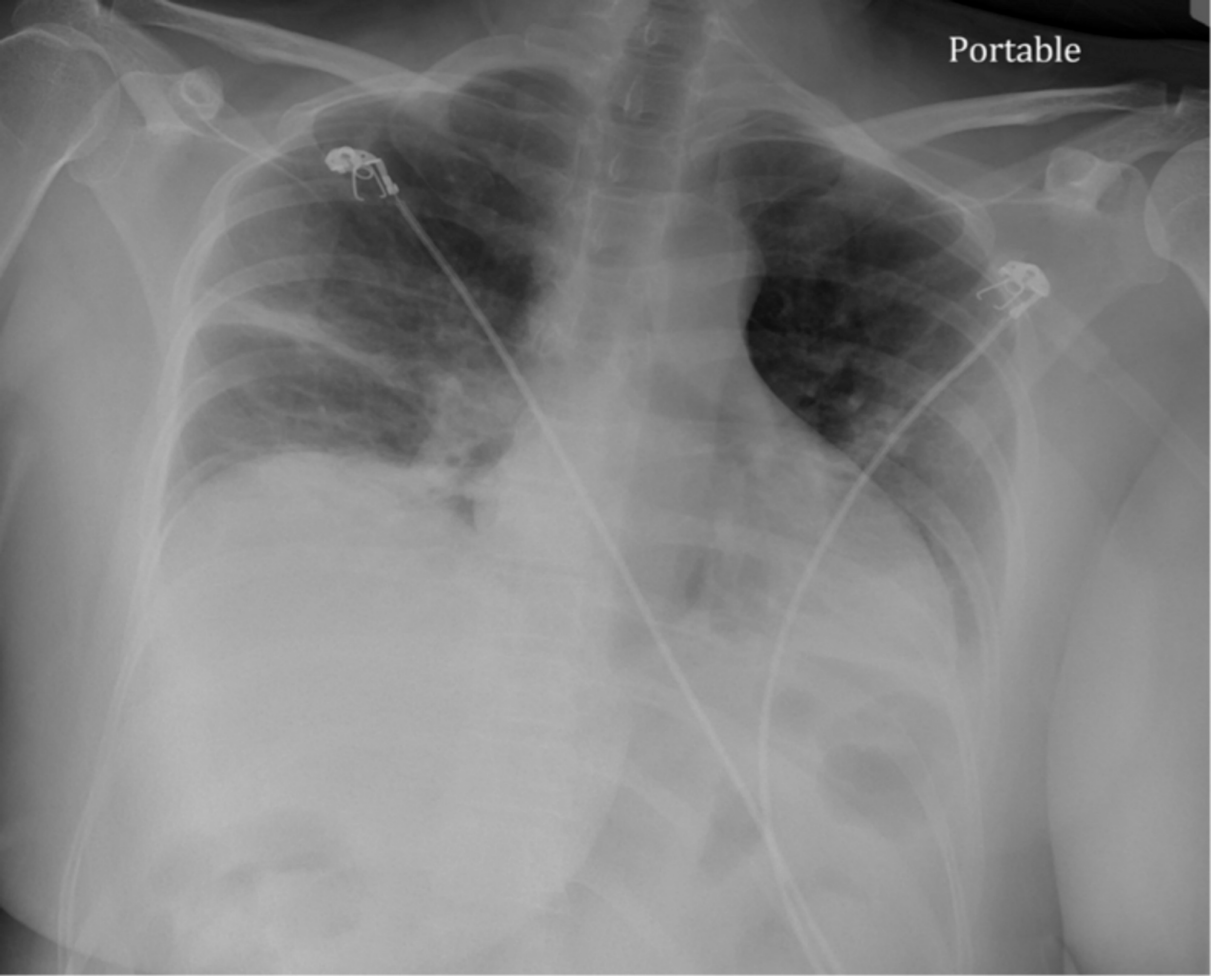
Portable Chest X-Ray showed Bilateral pulmonary opacities predominantly in peripheral and basal distribution

**Figure 2:**
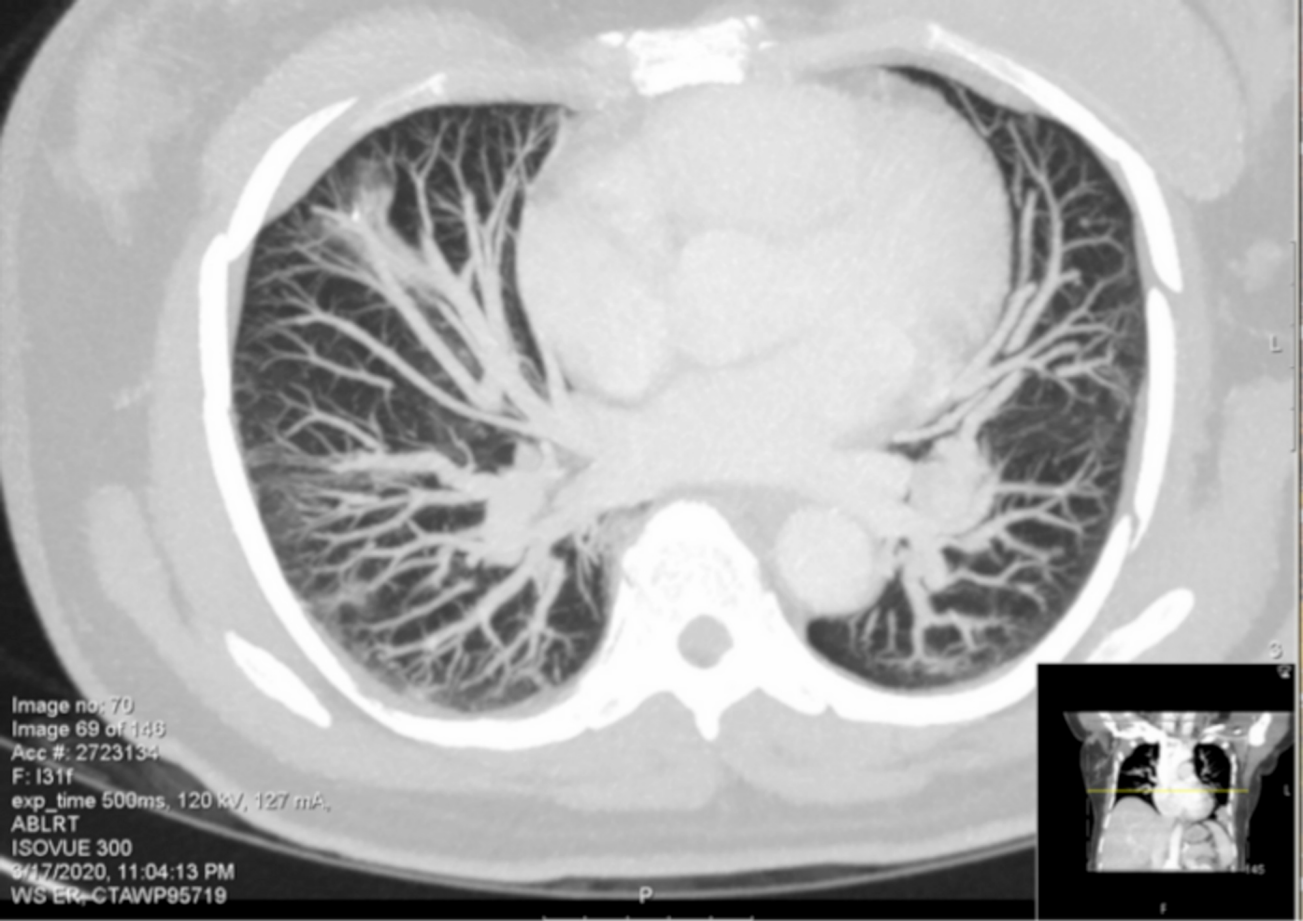
Chest CT showed bilateral patchy groundglass opacities predominantly in peripheral distribution

Arterial blood gas (ABG) showed pH 7.45, carbon dioxide partial pressure (pCO2) 39, oxygen partial pressure (pO2) 53 and O_2_ saturation 94.5% on 2L nasal cannula. Chest X-ray showed bilateral infiltrates as seen in Figure 1. CT chest showed multifocal patchy ground glass opacities predominantly in peripheral distribution, which was concerning for COVID-19 (Figure 2).

Influenza, respiratory syncytial virus (RSV), blood culture and urine culture were negative. White blood cell (WBC) was 4.7 K/UL (4.8-10.8 K/UL) with an absolute lymphocyte count 900 K/UL, platelet count 189 K (130-400 K), C-reactive protein (CRP) 1.9 ng/dl, point-of-care (POC) troponin 0.00 ng/ml, B-type natriuretic peptide (BNP) <15 pg/ml. Nasopharyngeal swab for COVID-19 was positive.

Management

Patient was started on azithromycin 500 mg daily and hydroxychloroquine 400 mg twice a day. Baseline electrocardiogram showed a QTc < 470 and remained normal for duration of treatment. Hydroxychloroquine was decreased to 200mg twice a day on day two as per protocol. 

**Figure 3:**
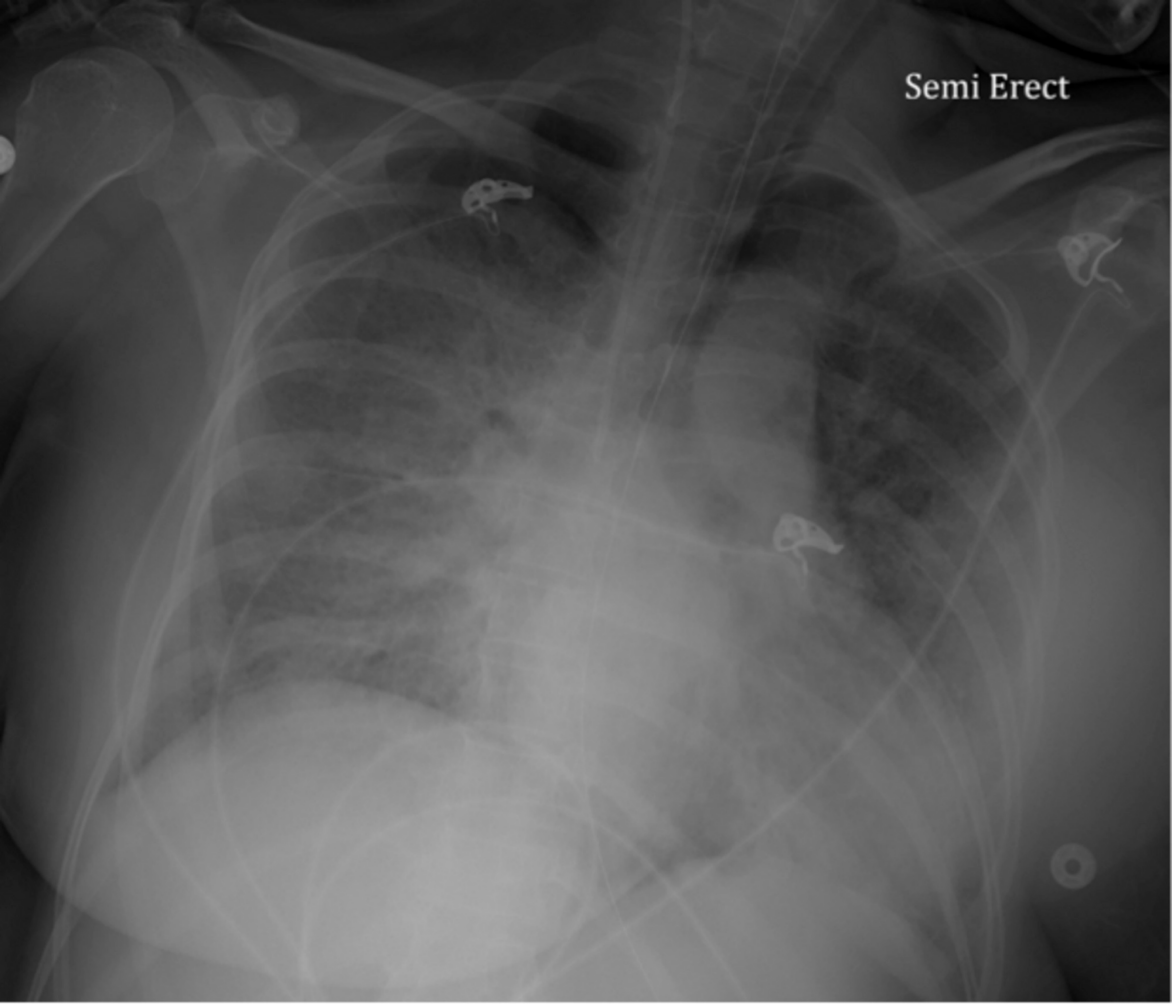
Chest X-ray showed progression of bilateral opacities with a more diffuse distribution.

Patient was maintained on nasal cannula and non-rebreather (NRB) with saturation between 92-96%. She suddenly decompensated on day six and was found to be in respiratory distress with oxygen saturation 50% on NRB and was unable to maintain saturation above 60% even on high flow resulting in decision to intubate. Post intubation, ABG showed pH 7.44, pCO2 41, pO2 31.8, which improved to pO2 90 on 100% fraction of inspired oxygen (FiO2) and positive end-expiratory pressure (PEEP) of 15. D-dimer and ferritin were elevated at >5000 ng/ml (0-230 ng/ml) and 2000 ng/ml (23.9-336.2 ng/ml), respectively. Chest X-ray showed diffuse bilateral airspace opacities, more pronounced than prior study as seen in Figure 3.

EKG showed sinus tachycardia. Patient became hypotensive a few hours later and was started on norepinephrine and phenylephrine. Propofol was switched to fentanyl and versed. 

**Figure 4:**
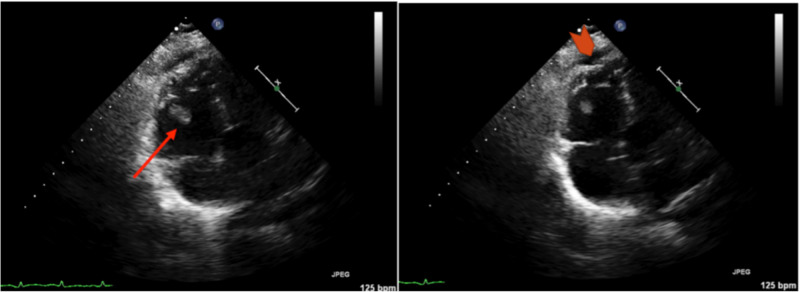
Trans thoracic echocardiogram showed right ventricular (RV) thrombus (red arrow) and McConnell’s sign with akinesia of the mid free wall of RV but normal motion at the apex (arrowhead)

An echocardiogram was ordered, and the patient was empirically started on therapeutic heparin. Of note, patient was on prophylactic dose of low molecular weight heparin (LMWH) (40 mg/day) since admission. Echocardiogram showed a large right ventricular (RV) echodensity, concerning for thrombus and McConnell’s sign (Figure 4) concerning for pulmonary embolism (PE).

**Figure 5:**
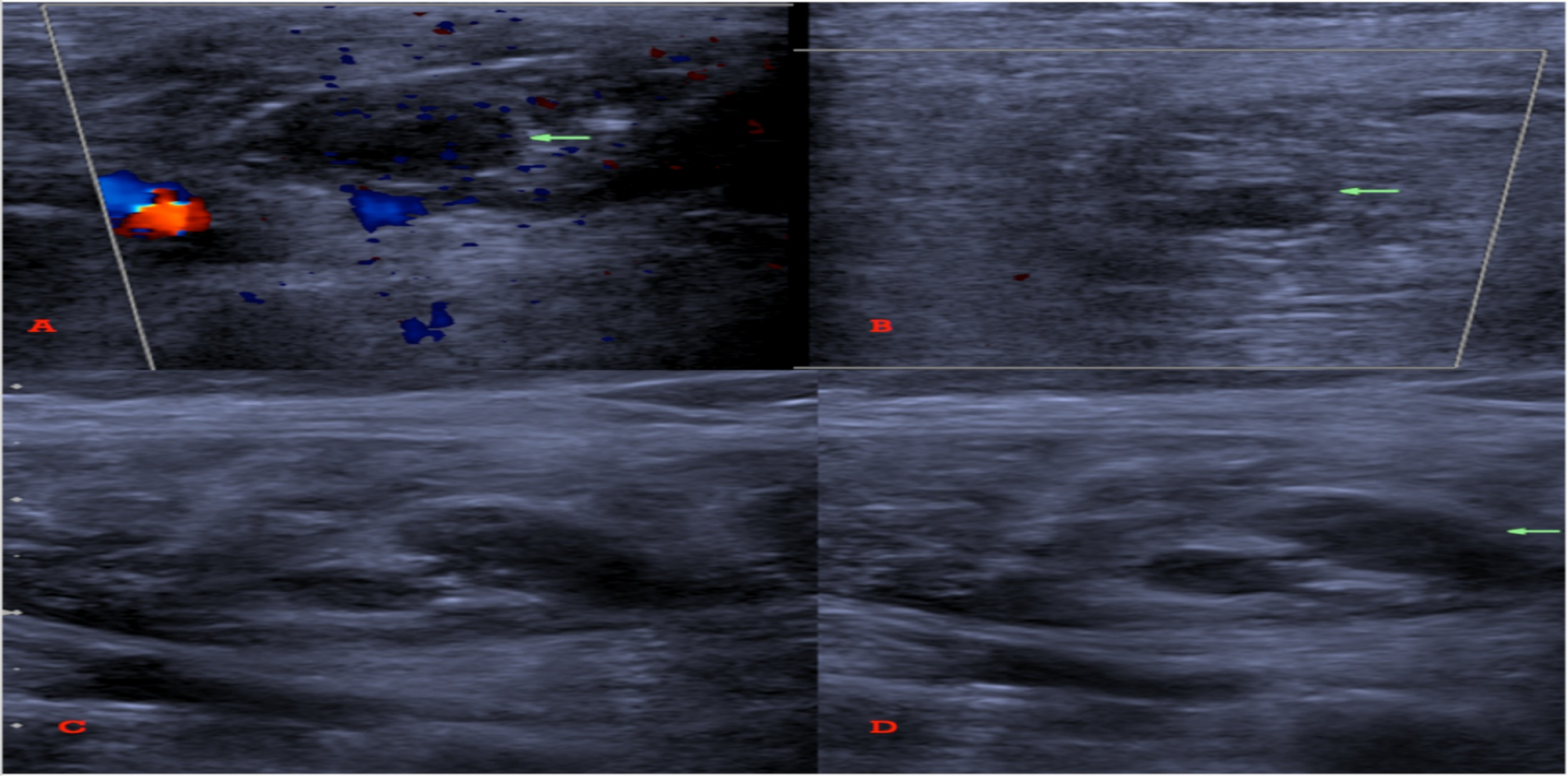
Lower extremity venous duplex showed (A) Right and (B) left intramuscular vein thrombosis. (C) Gastrocnemius vein without and (D) with compression showing non compressible vein indicative of thrombosis.

The patient was not considered a candidate for tissue plasminogen activator (tPA) based on the size of RV thrombus. Venous duplex showed bilateral lower calf venous thrombosis (Figure 5).

Patient continued to be ventilator-dependent, requiring high FiO2 and was started on hydrocortisone 100 mg i.v. every eight hours. Patient responded well to steroids showing improvement in oxygenation and blood pressure and was taken off pressor support. Patient’s volume status was closely monitored as she was on high PEEP for acute respiratory distress syndrome which could be detrimental in the setting of PE which is preload dependent.

## Discussion

Deep vein thrombosis and/or pulmonary embolism have been reported in patients with severe acute respiratory syndrome (SARS) coronavirus (CoV)1 previously [1,2]. Because SARS-CoV-2 shares 70-80% genetic similarity with SARS-CoV-1 [3], there might be mechanisms causing venous thrombosis similar to that in our patient. The interaction between the SARS viruses (SARS-CoV-1 and SARS-CoV-2) and angiotensin-converting enzyme 2 (ACE2) has been proposed as a potential factor in their infectivity and the expression of ACE2 in multiple systems including type II alveolar cells in the lungs, endothelium, kidneys and heart [4] is responsible for multisystem organ failure. This epithelial expression along with the presence of ACE2 in vascular endothelium has been hypothesized for the causation of inflammation and intravascular thrombosis, which were seen on autopsy studies [5]. D-dimer is a fibrin split product and has been used extensively as a marker for underlying thrombosis.

A retrospective cohort study identified several risk factors for death in adults in Wuhan hospitalized with COVID-19. The odds of dying in the hospital increased with age (odds ratio 1.10; 95% confidence interval, 1.03-1.17; per year increase in age), higher Sequential Organ Failure Assessment (SOFA) score (5.65, 2.61-12.23; P< .0001), and D-dimer level exceeding 1 mcg/L on admission [6]. The SOFA assesses rate of organ failure in intensive care units. D-dimer levels were also found to be higher among non-survivors than survivors [6] with a temporal increase in D-dimer with severity of illness. About 90% of inpatients with pneumonia had increased coagulation activity, marked by increased D-dimer concentrations [7] and D-dimer greater than 1 mcg/L was found to be associated with fatal outcome of COVID-19 and considered an independent risk factor of mortality. The administration of heparin in severe COVID-19 has been recommended by expert consensus from China [8].

Severe COVID-19 was defined as meeting any of the following criteria, according to the diagnosis and treatment plan of COVID-19 suggested by the National Health Commission of China: Respiratory rate ≥ 30breaths/min; arterial oxygen saturation ≤ 93% at rest; PaO_2_/FiO_2_ ≤ 300mmHg [9]. A recent retrospective study demonstrated no difference in 28-day mortality between heparin users and nonusers (30.3% vs 29.7%, P=0.910). But the 28-day mortality of heparin users was significantly lower than non-users in patients with sepsis-induced coagulopathy score (SIC) ≥4 (40.0% vs 64.2%, P=0.029) or D-dimer >3 mcg/L or >6 times the upper limit of normal (ULN) (32.8% vs 52.4%, P=0.017) [8]. In this study prophylactic doses of mostly LMWH (40-60 mg/day) and in some cases unfractionated heparin (UFH) (10000-15000 U/day) was used for seven or more days. Our patient had an elevated D-dimer >5 mcg/L and severe COVID-19. She was on prophylactic doses of LMWH (40 mg/day) prior to her sudden deterioration and was found to have extensive thrombosis.

## Conclusions

Even with recommendations based on expert consensus for prophylactic doses of LMWH or UFH, our patient without prior history of underlying hypercoagulable state developed extensive thrombosis despite being on prophylactic LMWH. Pulmonary thrombi, whether micro or macro, may be playing a significant role in mortality of some patients. Therapeutic heparin anticoagulation early in the course of the disease may be beneficial beyond those with clinically documented venous thromboembolism including those with severe COVID-19, elevated D-dimer >1 mcg/l on admission which is considered an independent predictor of mortality, or patients with temporal changes in D-dimer with increasing severity of disease.
